# Differential impact of 5-lipoxygenase-activating protein antagonists on the biosynthesis of leukotrienes and of specialized pro-resolving mediators

**DOI:** 10.3389/fphar.2023.1219160

**Published:** 2023-08-23

**Authors:** Philipp Dahlke, Lukas K. Peltner, Paul M. Jordan, Oliver Werz

**Affiliations:** ^1^ Department of Pharmaceutical/Medicinal Chemistry, Institute of Pharmacy, Friedrich Schiller University, Jena, Germany; ^2^ Jena Center for Soft Matter (JCSM), Friedrich Schiller University Jena, Jena, Germany

**Keywords:** 5-lipoxygenase-activating protein, 15-lipoxygenase, lipid mediators, leukotrienes, specialized pro-resolving mediators, lipoxins, macrophages, metabololipidomics

## Abstract

Lipoxygenases (LOX) transform arachidonic acid (AA, C20:4) and docosahexaenoic acid (DHA, C22:6) into bioactive lipid mediators (LMs) that comprise not only pro-inflammatory leukotrienes (LTs) but also the specialized pro-resolving mediators (SPMs) that promote inflammation resolution and tissue regeneration. The 5-LOX-activating protein (FLAP) is known to provide AA as a substrate to 5-LOX for generating LTs, such as LTB_4_, a potent chemoattractant and activator of phagocytes. Notably, 5-LOX is also involved in the biosynthesis of certain SPMs, namely, lipoxins and D-resolvins, implying a role of FLAP in SPM formation. FLAP antagonists have been intensively developed as LT biosynthesis inhibitors, but how they impact SPM formation is a matter of debate. Here, we show that FLAP antagonism suppresses the conversion of AA by 5-LOX to LT and lipoxins, while the conversion of DHA to SPM is unaffected. Screening of multiple prominent FLAP antagonists for their effects on LM formation in human M1- and M2-monocyte-derived macrophages by comprehensive LM profiling showed that all nine compounds reduced the production of 5-LOX-derived LTs but increased the formation of SPMs from DHA, e.g., resolvin D5. Some FLAP antagonists, especially those that contain an indole or benzimidazole moiety, even elicited SPM formation in resting M2-monocyte-derived macrophages. Intriguingly, in coincubations of human neutrophils and platelets that produce substantial AA-derived lipoxin and DHA-derived RvD5, FLAP antagonism abolished lipoxin formation, but resolvin D5 levels remained unaffected. Conclusively, antagonism of FLAP suppresses the conversion of AA by 5-LOX to LTs and lipoxins but not the conversion of DHA by 5-LOX to SPM, which should be taken into account for the development of such compounds as anti-inflammatory drugs.

## 1 Introduction

**FIGURE 1:**
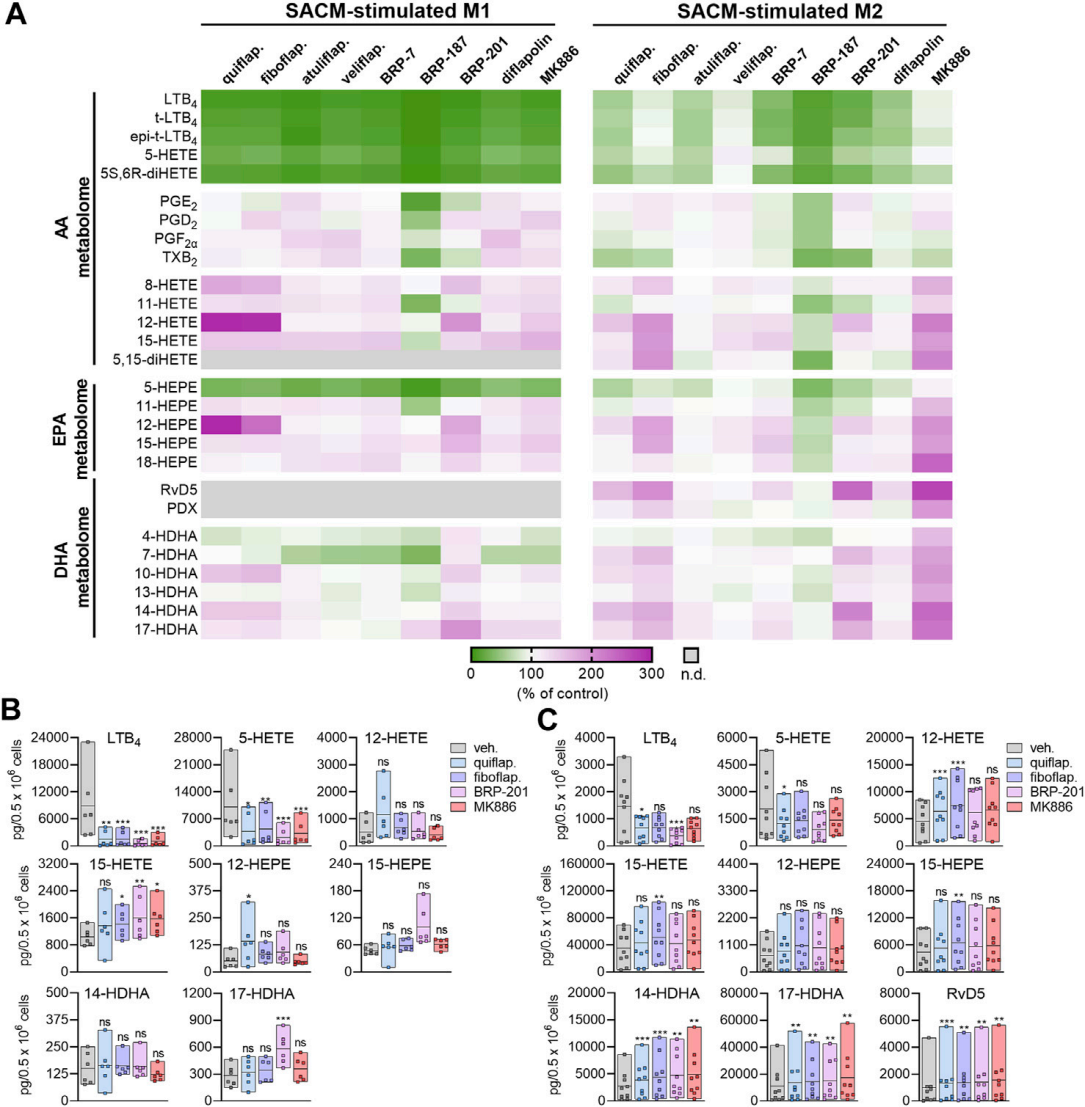
Modulation of agonist-induced lipid mediator formation by FLAP antagonists in MDMs. **(A–C)** Human M1- or M2-MDMs (0.5 × 10^6^ cells) were preincubated with the indicated FLAP antagonists: BRP-7 at 10 μM; quiflapon, fiboflapon, atuliflapon, and MK886 at 1 μM; and veliflapon, BRP-187, BRP-201, and diflapolin at 3 µM or with a vehicle (DMSO, 0.1%) for 15 min before the challenge with SACM (1%) for 90 min at 37°C. The formed LMs were quantified in the supernatants by UPLC–MS-MS. **(A)** Data (*n* = 3) are given as heatmaps presenting the fold change to the SACM-stimulated vehicle control. **(B,C)** Results for the selected LMs are shown for M1-MDMs **(B)** and M2-MDMs **(C)** treated with 1 µM quiflapon, fiboflapon, and MK886, 3 µM BRP-201, or with 0.1% vehicle (DMSO). Data (*n* = 6 for M1; *n* = 9 for M2) are given as pg LM per 0.5 × 10^6^ cells, shown as single values and as means in floating bar charts. For statistical analysis, data were log-transformed and analyzed via matched one-way ANOVA with Dunnett’s multiple comparison test; *p < 0.05, **p < 0.01, and ***p < 0.001; n.d., not detected.

The nuclear membrane-bound 5-lipoxygenase (LOX)-activating protein (FLAP) is devoid of enzymatic activity and assists 5-LOX in the two initial steps of leukotriene (LT) biosynthesis by provision of arachidonic acid (AA, C20:4) as a substrate (Evans et al., 2008; Ferguson et al., 2007). AA is first transformed by 5-LOX to 5(S)-hydroperoxyeicosatetraenoic acid (5-HpETE) and subsequently dehydrated to the 5,6-epoxide LTA_4_ (Radmark et al., 2015). LTA_4_ is then converted by LTA_4_ hydrolase to LTB_4_ or by LTC_4_ synthase, yielding the cysteinyl-LTs C_4_, D_4_, and E_4_ (Radmark et al., 2015) (Figure 1A). Based on the pivotal roles of LTs in inflammation and allergy, drugs interfering with either LT receptors (i.e., cysLT1) or with their biosynthesis have been developed for the treatment of asthma, allergies, arthritis, and cardiovascular disease (Haeggstrom, 2018). The latter encompass inhibitors of 5-LOX (e.g., zileuton), LTA_4_ hydrolase, and LTC_4_ synthase, as well as antagonists of FLAP (Werz et al., 2017). 5-LOX inhibitors and FLAP antagonists block the formation of both cysLTs and LTB_4_ and, apparently, all other 5-LOX-derived LMs (Gilbert et al., 2021). Only the 5-LOX-inhibitor zileuton (Zyflo^®^) has been approved for pharmacotherapy of asthma (in the US), while FLAP antagonists are not yet on the market, despite intensive development (Gur et al., 2018a; Prescott et al., 2022).

5-LOX, mainly expressed in myeloid cells, is a soluble protein located in the cytosol or nucleosol, depending on the cell type and environmental impact (Radmark et al., 2015). Upon adequate cell activation, 5-LOX translocates to the nuclear envelope, where AA is liberated by phospholipases (PLs) A_2_ and then provided by FLAP to 5-LOX for conversion; in fact, FLAP is essential for cellular LT biosynthesis (Evans et al., 2008; Gilbert et al., 2021). In contrast, the activities of 12/15-LOX are not affected by FLAP. In addition to AA, 5-LOX accepts other polyunsaturated fatty acid (PUFA) substrates that contain a cis, cis-1,4-pentadiene moiety and, thus, also converts eicosapentaenoic acid (EPA, C20:5) and docosahexaenoic acid (DHA, C22:6) into bioactive lipid mediators (LMs), including the more recently discovered specialized pro-resolving mediators (SPMs) that contribute to the termination and resolution of inflammation and promote tissue regeneration (Serhan, 2014; Serhan and Levy, 2018). The 5-LOX-dependent SPMs not only include AA-derived lipoxins (LX) A_4_ and B_4_ but also EPA-derived (E-series) resolvins (Rvs) E1, E2, and E4, as well as DHA-derived (D-series) RvD1-6 (Chiang and Serhan, 2020). The biosynthesis of LXs and RVs seemingly requires the dual action of 5-LOX and a 12-/15-LOX isoform (D-RVs) or CYP450 enzymes (E-RVs) (Chiang and Serhan, 2020).

The involvement of 5-LOX in SPM biosynthesis raises the question of whether FLAP is also needed for SPM formation and if FLAP also provides EPA and DHA, as well as monohydroxylated SPM precursors (e.g., 15(S)-HETE, 15(S)-HEPE, and 17(S)-HDHA) to 5-LOX for conversion (Gilbert et al., 2021; Kahnt et al., 2023). There are contradicting findings on the role of FLAP in SPM formation: activated 5-LOX in the cytosol, distant from FLAP, may receive *de novo*-generated 15-HETE by 15-LOX for conversion to LXA_4_ (Fredman et al., 2014), suggesting that 15-LOX-formed monohydroxylated precursors might be shuttled to 5-LOX independent of FLAP. Furthermore, DHA might be transformed by 5-LOX without FLAP, supported by the inability of the FLAP antagonists MK886 and BRP-201 to impair RvD5 formation in human M2 macrophages despite the suppression of LT biosynthesis (Werz et al., 2018; Werner et al., 2019; Kretzer et al., 2022a). In contrast, FLAP was found to be required for the generation of RvD1 and RvD5, in addition to RvE1, 5,15-diHETE, and LXA_4_, since their formation in neutrophils was blocked by MK-886 (Lehmann et al., 2015; Mainka et al., 2022).

Evaluation and mechanistic investigations of FLAP antagonists had been essentially performed in neutrophils or monocytes, focusing on AA-derived LTs, mainly neglecting the effects on SPM production and DHA/EPA transformation (Werz et al., 2017; Gur et al., 2018a; Gilbert et al., 2021). Moreover, previous cellular studies with FLAP antagonists applied cell-based test systems using short-term incubations (5–15 min) of leukocytes or cell lines that were stimulated with Ca^2+^-ionophore A23187, where PUFA or SPM precursors (e.g., 17-HDHA) were sometimes added exogenously (Lehmann et al., 2015; Mainka et al., 2022). Here, we studied the role of FLAP in SPM formation employing a comprehensive pharmacological approach in pathophysiological assay systems where endogenous PUFA substrates are transformed, monitoring a broad spectrum of AA-, EPA-, and DHA-derived LMs. Thus, we used human MDMs with either an M1- or M2-like phenotype, which we exposed to exotoxins from *Staphylococcus aureus* for 90 min (Werz et al., 2018; Werner et al., 2019; Jordan et al., 2020). M1-MDMs express abundant 5-LOX and FLAP and, upon exotoxin stimulation, generate LM profiles where LTs dominate, while M2-MDMs express 15-LOX-1 and 5-LOX but hardly FLAP, producing less LTs but DHA-derived SPMs instead (Werz et al., 2018; Radmark, 2022). Moreover, we exploited coincubations of human neutrophils and platelets that are known to produce substantial AA-derived LTs and LXs by transcellular metabolism (Serhan et al., 1999; Capra et al., 2015) for studying the effects of FLAP antagonists on LM profiles and the role of FLAP in SPM biosynthesis.

## 2 Materials and methods

### 2.1 Isolation of human leukocytes and platelets

Leukocyte concentrates obtained from freshly withdrawn blood (containing 16 I.E. heparin/mL blood) of healthy adult male and female volunteers (18–65 years) were provided by the Department of Transfusion Medicine at the University Hospital of Jena, Germany. The experimental procedures were approved by the local ethical committee of the University Hospital of Jena (Jena, Germany; approval no. 5050–01/17) and were performed in accordance with the respective guidelines and regulations. Written informed consent was obtained from the volunteers. According to previously published procedures (Pace et al., 2017), neutrophils and peripheral blood mononuclear cells (PBMCs) were isolated by density gradient centrifugation using a lymphocyte separation medium (C-44010, PromoCell, Heidelberg, Germany) after sedimentation of erythrocytes using dextran. Platelet-enriched plasma was collected from the supernatant after density gradient centrifugation, diluted with PBS pH 5.9 (4:1 *v/v*), and centrifuged (2,100 × g, 15 min, room temperature). The pelleted platelets were resuspended in a 1:1 (*v/v*) mixture of PBS pH 5.9 and NaCl solution (0.9% m/v) and washed two more times. Finally, the platelets were resuspended in PBS pH 7.4 containing CaCl_2_ (1 mM). For isolation of monocytes, PBMCs were seeded in PBS pH 7.4 containing CaCl_2_ and MgCl_2_ (Sigma-Aldrich, Steinheim, Germany) in cell culture flasks (Greiner Bio-One, Frickenhausen, Germany). After 1 h at 37°C and 5% CO_2_ for monocyte adherence, the medium was discarded and replaced with RPMI 1640 (Thermo Fisher Scientific, Schwerte, Germany), containing heat-inactivated fetal calf serum (FCS, 10% *v/v*), penicillin (100 U/mL), streptomycin (100 μg/mL), and L-glutamine (2 mmol/L).

### 2.2 Macrophage differentiation and polarization

Differentiation of monocytes to macrophages and subsequent polarization to M1- and M2-like phenotypes were carried out as described previously (Werz et al., 2018). In brief, PBMCs were incubated with either 20 ng/mL GM-CSF or M-CSF (Cell Guidance Systems Ltd., Cambridge, United Kingdom) for 6 days in RPMI 1640 supplemented with 10% FCS, L-glutamine, penicillin, and streptomycin. The obtained M0_GM-CSF_ MDMs were treated with LPS (100 ng/mL) and IFN-γ (20 ng/mL; PeproTech, Hamburg, Germany) for 24 h to obtain M1-MDMs, whereas and M0_M-CSF_ were treated with IL-4 (20 ng/mL; PeproTech) to generate M2-MDMs within 48 h.

### 2.3 Cell viability assays

For analyzing the effects of the FLAP antagonists on cell viability, cells were incubated with 3-(4,5-dimethylthiazol-2-yl)-2,5-diphenyltetrazolium bromide (MTT, 5 mg/mL, 20 μL; Sigma-Aldrich, Munich, Germany) for 2–3 h at 37°C (5% CO_2_) in the dark. The formazan product was solubilized with sodium dodecyl sulfate (SDS, 10% in 20 mM HCl), and the absorbance was monitored at 570 nm (Multiskan Spectrum microplate reader, Thermo Fisher Scientific, Schwerte, Germany). Staurosporine (1 µM) was used as the positive control. For analyzing the effects of the FLAP antagonists on cell integrity, the release of lactate dehydrogenase (LDH) was assessed using CytoTox 96^®^ Non-Radioactive Cytotoxicity assay according to the manufacturer´s instructions (Promega, Mannheim, Germany). After treatment, the cells were centrifuged at 400 g (5 min, 4°C), and the supernatants were diluted to suitable LDH concentrations. The absorbance was then measured at 490 nm using a NOVOstar microplate reader (BMG Lab Technologies GmbH, Offenburg, Germany). Cell integrity was calculated according to the manufacturer´s guidelines. Triton X-100 (0.9%, *v/v*) was used as the positive control.

### 2.4 Incubation for LM formation and LM metabololipidomics by UPLC–MS-MS

To study the effects of the FLAP antagonists on agonist-induced LM formation, M1- or M2-MDMs (0.5 × 10^6^/mL PBS containing 1 mM CaCl_2_) were incubated with a vehicle (DMSO, 0.1%) or FLAP antagonists for 15 min, prior to the addition of a *S. aureus*-conditioned medium (SACM) (1%) of the 6850 strain as a stimulus for 90 min at 37°C and 5% CO_2_. SACM was produced as previously described (Miek et al., 2022). To investigate the induction of LM formation by FLAP antagonists, the cells were incubated with the test compounds for 90 min at 37°C and 5% CO_2_ under the same conditions but without the addition of a stimulus. Afterward, the reaction was stopped by the addition of 2 mL of ice-cold methanol containing deuterated LM standards (200 nM d8-5S-HETE, d4-LTB_4_, d5-LXA_4_, d5-RvD2, d4-PGE_2_, and 10 µM d8-AA; Cayman Chemical/Biomol GmbH, Hamburg, Germany). The samples were kept at −20°C for at least 60 min to allow protein precipitation. The extraction of LMs was performed as previously described (Werz et al., 2018). In brief, after centrifugation (1,200 × g; 4°C; 10 min), acidified H_2_O (9 mL; final pH = 3.5) was added, and samples were extracted on solid phase cartridges (Sep-Pak^®^ Vac 6 cc 500 mg/6 mL C18; Waters, Milford, MA, United States). After equilibration of the cartridges with methanol, followed by H_2_O, the samples were loaded, with subsequent washing using H_2_O and *n*-hexane. LMs were eluted with methyl formate (6 mL). The solvent was fully evaporated using an evaporation system (TurboVap LV, Biotage, Uppsala, Sweden), and the residue was resuspended in 150 µL methanol/water (1:1, *v/v*) for UPLC–MS-MS analysis. LMs were analyzed using an Acquity™ UPLC system (Waters, Milford, MA, USA) and a QTRAP 5500 Mass Spectrometer (ABSciex, Darmstadt, Germany) equipped with a Turbo V™ Source and electrospray ionization. LMs were eluted using an ACQUITY UPLC^®^ BEH C18 column (1.7 µm, 2.1 mm × 100 mm; Waters, Eschborn, Germany) heated at 50°C with a flow rate of 0.3 mL/min and a mobile phase consisting of methanol–water–acetic acid at a ratio of 42:58:0.01 (*v/v/v*) that was ramped to 86:14:0.01 (v/v/v) over 12.5 min and then to 98:2:0.01 (v/v/v) for 3 min. The QTRAP 5500 system was run in the negative ionization mode by scheduled multiple reaction monitoring (MRM) coupled with information-dependent acquisition. The scheduled MRM window was 60 s, and optimized LM parameters were adopted, with a curtain gas pressure of 35 psi. The retention time and at least six diagnostic ions for each LM were confirmed using an external standard for each and every LM (Cayman Chemical/Biomol GmbH). Quantification was achieved by calibration curves for each LM. Linear calibration curves were obtained for each LM and yielded *r* ^2^ values of 0.998 or higher. The limit of detection for each targeted LM was determined as described previously (Werner et al., 2019). For UPLC–MS-MS analysis, the limit of detection (LOD) was 3 pg/sample, and this value was taken to express the fold increase for samples where the LM was below the detection limit. Below the LOD, LMs were not detected (n.d.) and stated as such.

### 2.5 Subcellular localization of 5-LOX and 15-LOX-1 by immunofluorescence microscopy

M0_M-CSF_ MDM (0.5 × 10^6^ cells) were seeded onto glass coverslips in 12-well plates and polarized to M2-MDMs for 48 h. The cells were then washed, and PBS pH 7.4 containing CaCl_2_ (1 mM) was added and incubated with the FLAP antagonists, SACM [1%; as the positive control (Jordan et al., 2020)], or a vehicle (DMSO; 0.1%) for 90 min at 37°C and 5% CO_2_. The cells were subsequently fixed using 4% paraformaldehyde solution in PBS. Permeabilization was achieved by adding acetone (3 min; 4°C) and then triton X-100 (0.25% solution; 10 min; room temperature). Following permeabilization, the samples were blocked with normal goat serum (10%, 50062Z, Thermo Fisher Scientific) and then incubated with mouse monoclonal anti-15-LOX-1 antibody, 1:200 (ab119774, Abcam, Cambridge, United Kingdom) and rabbit anti-5-LOX antibody, 1:100 (1550 AK6, kindly provided by Dr. Olof Rådmark, Karolinska Institutet, Stockholm, Sweden), overnight at 4°C. 5-LOX and 15-LOX-1 were stained with the fluorophore-labeled secondary antibodies: Alexa Fluor 488 goat anti-rabbit IgG (H + L), 1:500 (A11034, Thermo Fisher Scientific), and Alexa Fluor 555 goat anti-mouse IgG (H + L), 1:500 (A21424, Thermo Fisher Scientific). Nuclear DNA was stained with ProLong Diamond Antifade Mountant with DAPI (P36971, Thermo Fisher Scientific). The samples were analyzed using a Zeiss Axiovert 200 M microscope and a Plan-Neofluar ×40/1.30 Oil (DIC III) objective (Carl Zeiss, Jena, Germany). An Axiocam MR camera (Carl Zeiss) was used for image acquisition.

### 2.6 Statistics

The results are expressed as the means ± standard error of the mean (SEM) of n observations, where n represents the number of experiments with separate donors, performed on different days, as indicated. Analyses of the data were conducted using GraphPad Prism 8 software (San Diego, CA). Matched one-way analysis of variance (ANOVA) for multiple comparisons with Dunnett´s *post hoc* tests was applied as indicated. The criterion for statistical significance was *p* < 0.05.

## 3 Results

### 3.1 Differential modulation of agonist-induced LOX product formation by FLAP antagonists in human MDMs

FLAP antagonists, regardless of the chemotype, are proposed to compete with the FLAP-mediated AA transfer to 5-LOX and to impede the assembly of the LT-biosynthetic 5-LOX/FLAP complex at the nuclear membrane upon cell activation (Evans et al., 2008). In order to reveal a defined and common pattern of LM biosynthesis modulation by FLAP antagonists, we selected nine prominent candidate compounds from major chemical classes (Table 1) for initial screening. MK886 (Gillard et al., 1989) and fiboflapon (Stock et al., 2011) are indole-based (I); veliflapon (Hatzelmann et al., 1993) and BRP-187 (Garscha et al., 2016) are quinoline-based (II); quiflapon (Brideau et al., 1992) is an indole–quinoline hybrid (III); and BRP-7 (Pergola et al., 2014) and BRP-201 (Gur et al., 2018b) are benzimidazoles (IV), while diflapolin (Garscha et al., 2017) and atuliflapon (Pettersen et al., 2019) have unique chemical structures (V) (Table 1). M1- and M2-MDMs were preincubated (15 min) with these FLAP antagonists at two reasonable effective concentrations (low conc.–high conc.) each (Table 1), aiming at suppressing LT formation by much less than 50% or approximately 50%, respectively, according to published data (Gillard et al., 1989; Brideau et al., 1992; Hatzelmann et al., 1993; Stock et al., 2011; Pergola et al., 2014; Garscha et al., 2016; Garscha et al., 2017; Gur et al., 2018b; Pettersen et al., 2019). The cells were then exposed to 1% SACM containing LOX-stimulatory exotoxins (Jordan et al., 2020), and after 90 min at 37°C, the formation of up to 27 LMs was assessed by comprehensive LM metabololipidomics by UPLC–MS-MS (Werner et al., 2019).

**TABLE 1:** Overview of the investigated FLAP antagonists with the used concentrations and reported IC_50_ values in leukocyte-based assays.

Class	Compound	Chemical structure	Concentration used		IC_50_ [nM]	Reference
I	MK886	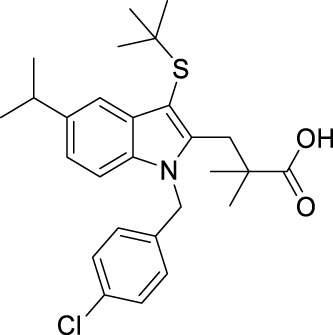	Low [µM]	High [µM]		
			0.03	1	2.5	Gillard et al. (1989)
	Fiboflapon	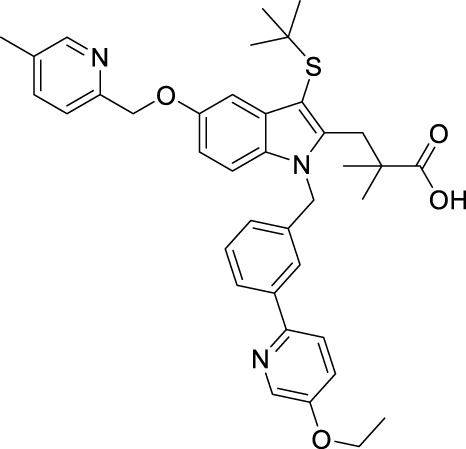	0.03	1	0.7	Stock et al. (2011)
II	BRP-187	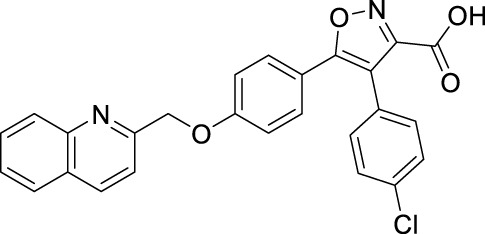	0.3	3	60	Garscha et al. (2016)
	Veliflapon	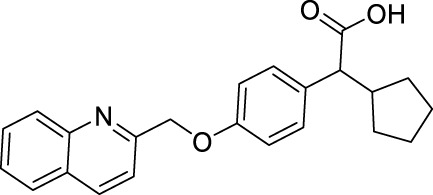	0.3	3	220	Hatzelmann et al. (1993)
III	Quiflapon	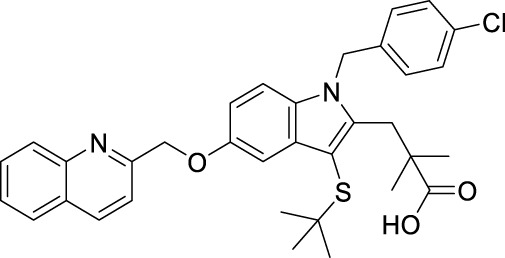	0.03	1	3.1	Brideau et al. (1992)
IV	BRP-7	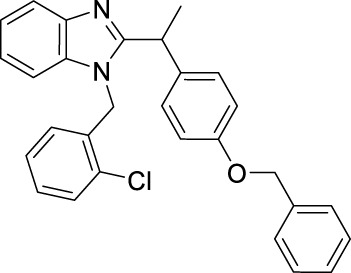	1	10	150	Pergola et al. (2014)
	BRP-201	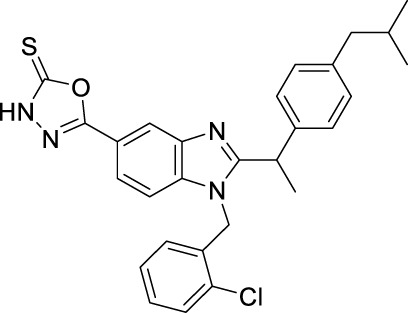	0.3	3	50	Gur et al. (2018b)
V	Diflapolin	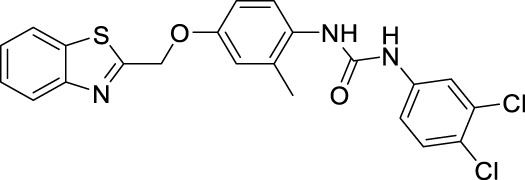	0.3	3	170	Garscha et al. (2017)
	Atuliflapon	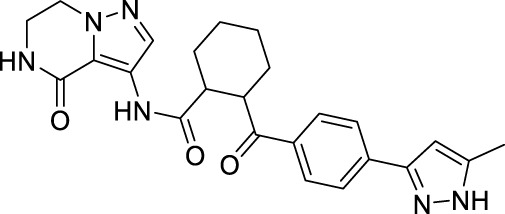	0.03	1	2	Pettersen et al. (2019)

In M1-MDMs that markedly express 5-LOX and FLAP but not or hardly 15-LOXs (Werz et al., 2018; Werner et al., 2019), all nine FLAP antagonists (at high conc.) efficiently suppressed the formation of 5-LOX products derived from AA and EPA by >50%, as expected, without marked differences between the individual 5-LOX-derived LMs LTB_4_, t-LTB_4_, et-LTB_4_, 5-HETE, 5S,6R-diHETE, and 5-HEPE (Figure 1A). Notably, the generation of 7-HDHA, the DHA-derived analog of AA/EPA-derived 5-HETE/5-HEPE formed by 5-LOX, was much less or not at all suppressed. Furthermore, as expected, the FLAP antagonists, except BRP-187 and veliflapon, did not inhibit 12/15-LOX product formation, with rather increasing features, especially for the indole-based fiboflapon and quiflapon that markedly increased 12-HETE and 12-HEPE levels. More detailed analysis of the effects of MK886, fiboflapon, quiflapon, and BRP-201 as the most prominent representatives on the selected 5-LOX and 15-LOX products showed that all four compounds potently inhibited the generation of LTB_4_, 5-HETE, and 5-HEPE but not 7-HDHA, all of which are 5-LOX products, while the formation of 15-LOX-derived 15-HETE, 15-HEPE, and 17-HDHA was not impaired (Figures 1A, B).

In M2-MDMs that substantially express 5-LOX and 15-LOX-1 but less FLAP (Werz et al., 2018; Werner et al., 2019), inhibition of 5-LOX product formation from AA and EPA by the FLAP antagonists was less pronounced, with fiboflapon and veliflapon being inactive, and the generation of 7-HDHA was even increased (Figures 1A, C). Again, as observed for M1-MDMs, the formation of 12/15-LOX products in M2-MDMs was not markedly suppressed by the FLAP antagonists (except for BRP-187 and veliflapon) but, rather, increased, regardless of the PUFA substrate (Figures 1A, C). Interestingly, the FLAP antagonists significantly increased the levels of DHA-derived RvD5, an SPM proposed to be formed by the concomitant action of 5-LOX and 15-LOX (Werner et al., 2019; Kahnt et al., 2023) (Figure 1C).

Neither in M1- nor M2-MDM the formation of COX products from AA and EPA (PGE_2_, PGD_2_, PGF_2_α, TXB_2_, 11-HETE, and 11-HEPE) was markedly affected by the FLAP antagonists, except for BRP-187 with inhibitory activity, especially for PGE_2_ in M1-MDMs (Figure 1A). Originally, BRP-187 was developed as a dual FLAP/mPGES-1 inhibitor, and thus, in addition to FLAP, mPGES-1 is also suppressed by BRP187 (Garscha et al., 2016), which may explain the slightly decreased PGE_2_ levels. Such dual FLAP/mPGES-1 inhibitors also act on mPGES-1, but compared to the suppression of FLAP-dependent 5-LOX product formation, they are less potent to reduce PGE_2_ formation and may require higher concentrations in this respect, especially in intact cells (Werz et al., 2017). Interestingly, BRP-187 and BRP-201 suppressed TXB_2_ formation in M1- and M2-MDMs, which was not observed by other FLAP antagonists studied and might be due to the modulation of COX or TXAS enzymes.

Similar patterns of LM modulation in M1- and M2-MDMs by these nine FLAP antagonists were observed at lower concentrations with overall somewhat low magnitudes of inhibition or stimulation, depending on the individual compound (Supplementary Figure S1). Moreover, none of the compounds at the tested concentrations displayed detrimental effects on MDM membrane integrity within 3 h, assessed by LDH release, or on metabolic activity within 48 h, assessed by MTT assay (Supplementary Figure S2). Together, the data suggest divergent actions of the FLAP antagonists on LOX product formation depending on the PUFA substrates, i.e., a common pattern for AA and EPA but different for DHA.

### 3.2 Induction of LOX product formation by FLAP antagonists in M2-MDMs

**FIGURE 2:**
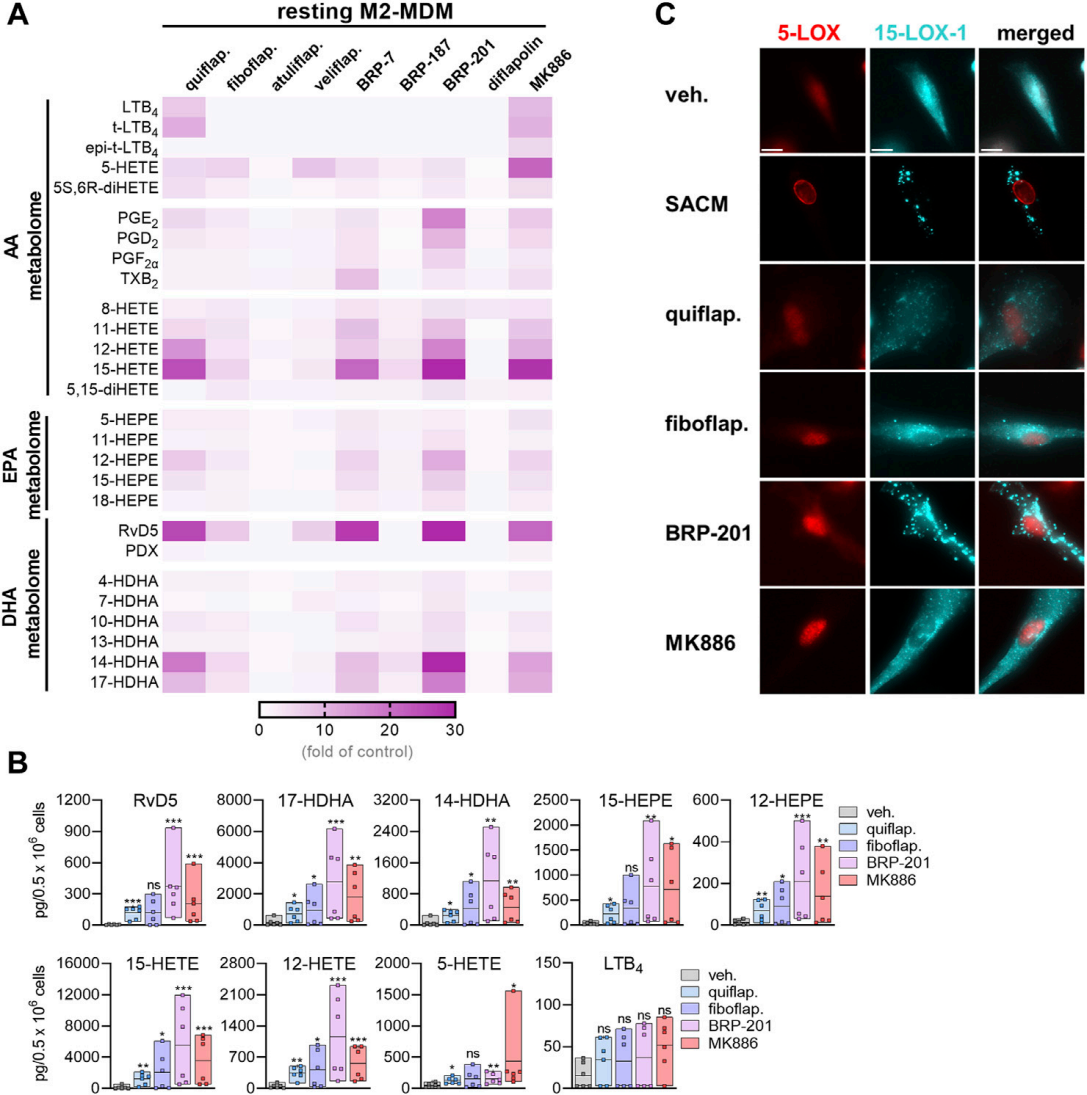
Induction of LM formation by FLAP antagonists in M2-MDMs. **(A)** Human M2-MDMs (0.5 × 10^6^ cells) were incubated with FLAP antagonists: BRP-7 at 10 μM; quiflapon, fiboflapon, atuliflapon, and MK886 at 1 μM; and veliflapon, BRP-187, BRP-201, and diflapolin at 3 µM or with vehicle (DMSO, 0.1%) for 180 min at 37°C. Formed LMs were quantified in the supernatants by UPLC–MS-MS. **(A)** Results are given as a heatmap presenting the fold change to the vehicle control; *n* = 3. **(B)** Selected LMs produced in M2-MDMs upon stimulation with 1 µM quiflapon, fiboflapon, and MK886, 3 µM BRP-201, or with 0.1% vehicle for 180 min given as single values and as means in floating bar charts; *n* = 6 independent experiments. For statistical analysis, data were log-transformed and analyzed via matched one-way ANOVA with Dunnett’s multiple comparison test. *p < 0.05, **p < 0.01, and ***p < 0.001. **(C)** M2-MDMs were incubated with 1% SACM, 1 µM quiflapon, 1 µM fiboflapon, 3 µM BRP-201, or with vehicle (DMSO, 0.1%), and the incubation was stopped after 90 min at 37°C. Cells were fixed, permeabilized, incubated with antibodies against 5-LOX (red) and 15-LOX-1 (cyan blue), and analyzed by immunofluorescence microscopy; scale bar = 10 µm. Results, shown for a single cell, are representative for approximately 100 individual cells analyzed in *n* = 3 independent experiments.

Our previous study showed that the FLAP antagonist BRP-201 acts as a stimulus in resting M2-MDMs to induce LM formation, especially that of 12/15-LOX-derived SPMs (Kretzer et al., 2022a). Thus, we tested the FLAP antagonists in this respect in order to determine if such an induction of 12/15-LOX product formation is a class effect of these compounds. In contrast to SACM and A23187, we previously observed that other agents, such as boswellic acids (Borner et al., 2023), celastrol (Pace et al., 2021), or BRP201 (Kretzer et al., 2022a), require longer incubation periods (up to 180 min) to efficiently stimulate 12/15-LOX product (i.e., SPM) formation in MDMs. Therefore, MDMs were exposed for 180 min to the FLAP antagonist to study their ability to induce 12/15-LOX product formation. Upon incubation of M2-MDMs for 180 min with the FLAP antagonists at the higher concentrations used (Table 1), quiflapon, BRP-7, BRP-201, and MK886 were the most efficient and caused comparable stimulation of 12/15-LOX product generation with the most pronounced increase for 12-HETE, 15-HETE, RvD5, 14-HDHA, and 17-HDHA (Figures 2A, B). Fiboflapon, veliflapon, and BRP-187 were less efficient, and diflapolin and atuliflapon were essentially inactive. It should be noted that in resting (unstimulated) MDMs, quiflapon and MK-886 also stimulated the formation of 5-LOX products, including 5-HETE and LTB_4_ (Figure 2A; Supplementary Figure S3C). Intriguingly, those antagonists that strongly induced 12/15-LOX product formation caused subcellular redistribution of 15-LOX-1 (especially BRP-201), similar to that observed with SACM, although to a minor degree, while 5-LOX did not translocate (Figure 2C). Investigating all nine FLAP antagonists side by side with cells from the same donor is experimentally not possible; thus, only four representative compounds from three different chemical classes were employed. COX products were hardly or not induced by the FLAP antagonists, except for BRP-201 which particularly increased PGE_2_ formation. Upon incubation of M1-MDMs with the FLAP antagonists, no specific induction of 12/15-LOX products was observed, while 8-HETE was strongly increased (Supplementary Figures S3A, B). Together, these data indicate that activation of 15-LOX-1 for the generation of 12/15-lipoxygenated products is not a class effect of FLAP antagonists but, instead, depends on structural features and might be a FLAP-independent mechanism.

### 3.3 FLAP antagonists differentially modulate agonist-induced LOX product formation in stimulated neutrophil–platelet coincubations

**FIGURE 3:**
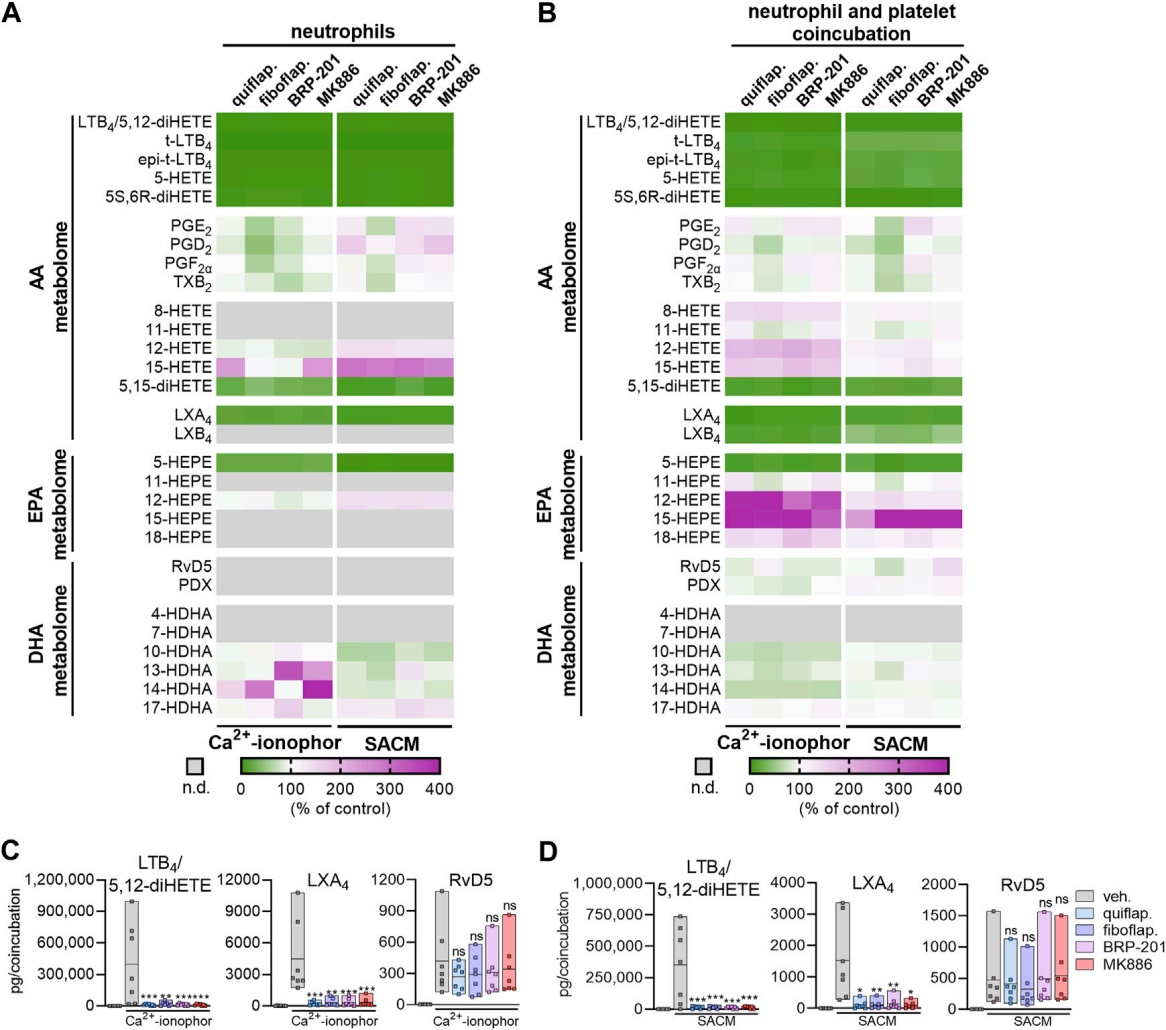
FLAP antagonists differentially modulate agonist-induced LOX product formation in stimulated neutrophil–platelet coincubations. Isolated neutrophils (1 × 10^7^ cells/mL) or coincubations of neutrophils and platelets (1 × 10^7^ and 25 × 10^7^ cells/mL, respectively) were incubated in PBS pH 7.4 containing 1 mM Ca^2+^ in the presence or absence of FLAP antagonists for 15 min. Then, SACM (1%) or Ca^2+^-ionophore A23187 (2.5 µM) was added, and incubation was continued for another 90 min or 15 min, respectively. **(A)** Results of stimulated neutrophils are given as a heatmap presenting the fold change to the vehicle control; *n* = 7. **(B)** Results of stimulated neutrophil–platelet coincubations are given as a heatmap presenting the fold change to the vehicle control; *n* = 7. **(C,D)** Results for selected LMs are shown as pg LM per coincubation as single values and as means in floating bar charts for **(C)** Ca^2+^-ionophore A23187-stimulated coincubations or **(D)** SACM-stimulated coincubations, *n* = 7. For statistical analysis, data were log-transformed and analyzed via matched one-way ANOVA with Dunnett’s multiple comparison test; *p < 0.05, **p < 0.01, and ***p < 0.001; n.d., not detected.

Coincubation of neutrophils, expressing 5-LOX, with platelets, expressing p12-LOX, accomplishes efficient LT and lipoxin formation by transcellular biosynthesis upon stimulation (Capra et al., 2015). We used SACM or Ca^2+^-ionophore A23187 as stimuli of such neutrophil–platelet coincubations, as well as of neutrophils alone for 90-min incubations. Both agents induce massive Ca^2+^ influx and may cause cytotoxic effects, especially after prolonged (>60 min) incubations. Nevertheless, the enzymatic LM biosynthetic machinery is primarily operative (Jordan et al., 2020), supported by the expected actions of FLAP antagonists that are evident only in viable cells (Kretzer et al., 2022a). The four FLAP antagonists MK886, fiboflapon, quiflapon, and BRP-201 were selected as tool compounds to study whether SPM formation in these cocultures is affected by FLAP antagonism as they displayed the most prominent effect on RvD5 formation in M2-MDMs; again, investigating all nine FLAP antagonists side by side with cells from the same donor is experimentally not possible. Neutrophils alone produced substantial amounts of 5-LOX products with either stimulus, especially with A23187, but A23187 elicited less 12/15-LOX products than SACM (Supplementary Table S1). All four FLAP antagonists efficiently blocked 5-LOX product formation but not 12/15-LOX and COX activities, as expected (Figure 3A).

In agreement with the literature (Capra et al., 2015), neutrophil–platelet coincubations robustly formed LTB_4_ and, in contrast to the neutrophils alone, also generated high amounts of the AA-derived LXA_4_ and LXB_4_, as well as DHA-derived RvD5 (Supplementary Table S1), which both require 5-LOX and a 12-LOX or 15-LOX isoform for their biosynthesis (Chiang and Serhan, 2020). As anticipated, the FLAP antagonists clearly blocked the formation of AA- and EPA-derived 5-LOX products, such as LTB_4_/5S,12S-diHETE, LTB_4_ trans-isomers, 5-HETE, 5S,6R-diHETE, and 5-HEPE in these coincubations, regardless of the stimulus (Figures 3B–D). Of note, the formation of LTB_4_ and/or 5S-12S-diHETE was extremely high (Supplementary Table S1), and due to the limitation of the resolution of the UPLC system with almost the same retention times of the respective peaks, these two LMs could not be separated and are, thus, referred to as the sum of LTB_4_ and 5S-12S-diHETE. Furthermore, formation of AA-derived LMs that require both 5-LOX and 12-/15-LOXs for their biosynthesis, such as LXA_4_ and LXB_4_, as well as 5,15-diHETE, was significantly reduced by all four FLAP antagonists. The chromatograms for LXA_4_ with two specific product ions are shown in Supplementary Figure S4. In sharp contrast, the FLAP antagonists failed to inhibit the formation of DHA-derived RvD5, which was also generated by the dual action of 5-LOX and 15-LOXs, again regardless of the stimulation with SACM or A23187 (Figures 3B–D). As expected, the FLAP antagonists, especially fiboflapon, increased AA- and EPA-derived 12/15-LOX products (about four-fold) when cells were stimulated with SACM or A23187. Altogether, these results suggest a differential requirement of FLAP for 5-LOX-mediated LM formation from AA/EPA and from DHA as substrates, which is further governed by the stimulus used to activate 5-LOX in the cell.

## 4 Discussion

**FIGURE 4:**
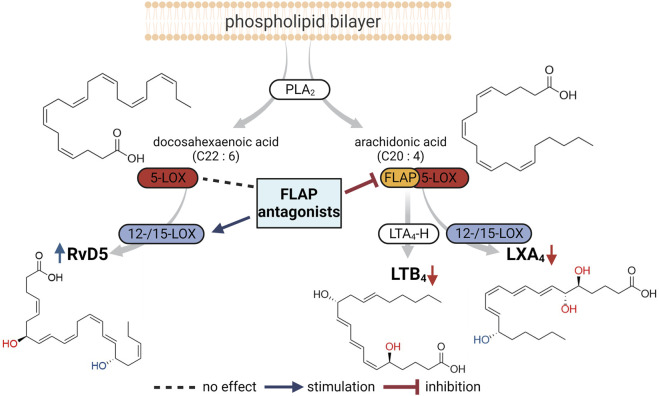
Overview of the proposed impact of FLAP antagonists on cellular LM networks. PLA_2_ enzymes liberate arachidonic acid (AA) and docosahexaenoic acid (DHA) as substrates for LOXs to produce LTB_4_ and LXA_4_ from AA and RvD5 from DHA. Formation of LTB_4_ and LXA_4_, involving 5-LOX, requires FLAP and, thus, is blocked by FLAP antagonists. RvD5 production by 5-LOX and 15-LOX occurs in the absence of FLAP, where some FLAP antagonists may even stimulate RvD5 formation.

The role of FLAP in SPM biosynthesis in leukocytes has been a matter of debate and is still obscure (Lehmann et al., 2015; Gilbert et al., 2021; Kahnt et al., 2023). Accordingly, the effect of FLAP antagonists on SPM formation and, thus, their benefit for clinical use in pharmacotherapy are unclear. The major SPMs formed from AA, EPA, or DHA are the LXs A_4_ and B_4_, RvE1-4, RvD1-6, protectins, and maresins (Chiang and Serhan, 2020). The production of protectins and maresins is 5-LOX-independent, but 5-LOX is seemingly involved in the formation of LXs, RvE1, RvE2, RvE4, and RvD1-6 (Chiang and Serhan, 2020), implicating that, in analogy to LT formation, FLAP is essential for their cellular production. In fact, experimental evidence using the FLAP antagonist MK886 as a chemical tool supports the necessity of FLAP for 5-LOX-mediated SPM (i.e., RvD5 and RvE1) biosynthesis in human neutrophils (Lehmann et al., 2015; Mainka et al., 2022). However, other studies using MK886 have concluded that in human macrophages, some SPMs, i.e., DHA-derived RVs, are generated in a FLAP-independent manner (Werz et al., 2018; Werner et al., 2019). Moreover, certain FLAP antagonists increased or even induced SPM formation in leukocytes (Kretzer et al., 2022a). Here, we employed a panel of nine prominent and structurally diverse FLAP antagonists to study the involvement of FLAP in the broad LM biosynthetic networks of pro- and anti-inflammatory human MDMs, neutrophils, and neutrophil–platelet coincubations. Our data obtained with these multiple FLAP antagonists propose that the requirement of FLAP for 5-LOX-mediated SPM formation depends on the type of PUFA substrate, where FLAP provides AA and EPA for LX and E-RVs, while DHA conversion to SPM by 5-LOX proceeds without FLAP, an overview of which is given in Figure 4.

In contrast to the results with FLAP inhibitors, which increase RvD5 production in M2-MDMs, 5-LOX inhibition by zileuton decreased RvD5 levels (Werner et al., 2019). This indicates that 5-LOX activity is required for producing the SPM RvD5, but FLAP is seemingly not needed. There are several reasons why the transformation of DHA by 5-LOX in MDMs and neutrophils could occur without the participation of FLAP. First, DHA in the cell might not sufficiently access and/or bind to FLAP. However, DHA was found to bind FLAP with even higher affinity versus AA in a FLAP antagonist-binding competition assay (Charleson et al., 1994). Second, transfer of DHA via FLAP to 5-LOX might not be favorable compared to the provision of AA (or EPA). While FLAP was indeed shown to stimulate conversion of AA by 5-LOX (Abramovitz et al., 1993), for DHA (and EPA), such data are not available. Third, DHA is released by a PLA_2_ other than AA and EPA, and thus, the subcellular locale of DHA liberation might be distant from FLAP that is located at the nuclear envelope. This is quite plausible in view of the two different PLA_2_ isoforms that preferably liberate AA and EPA, namely, cPLA_2_ and iPLA_2_, and DHA, namely, iPLA_2_-VIA and sPLA_2_ enzymes, and these different PLA_2_s exert their action on different PL pools during cellular activation (Hayashi et al., 2021).

In agreement with the findings of other studies (Lehmann et al., 2015; Mainka et al., 2022), our data show that FLAP is essential for LX formation in human neutrophils alone or in coincubation with platelets. Furthermore, in previous experiments using *E. coli*- or exotoxin-activated M2-MDMs, FLAP antagonism by MK886 decreased LXA_4_ formation but rather increased RvD5 levels (Werz et al., 2018; Werner et al., 2019). LX formation requires the dual action of 5-LOX and 12-/15-LOX (Kahnt et al., 2023), where two different sequential pathways are conceivable: 1) the 5-LOX:12/15-LOX route, where 5-LOX/FLAP first converts AA to LTA_4_ that is then transformed by 12/15-LOXs to LXs; or 2) the 12/15-LOX:5-LOX route, where 12/15-LOXs oxygenate AA to 15-H(p)ETE that is then converted by 5-LOX/FLAP to LXs. The second, more likely route, implies that FLAP would bind 15-H(p)ETE and hand it over to 5-LOX. Indeed, it was found that FLAP not only accepts AA but also assists 5-LOX in the conversion of 12(S)- and 15(S)-HETE to 5(S),12(S)- and 5(S),15(S)-diHETE, respectively (Hill et al., 1992), and LXA_4_ formation in human neutrophils from exogenously added 15-HETE was blocked by MK886 (Lehmann et al., 2015). On the other hand, *in vivo*, the FLAP antagonist veliflapon (syn. BAY X 1005) increased LXA_4_ levels in murine liver injury, while blocking cysLT formation (Titos et al., 2005).

In contrast to AA-derived LX formation, the generation of DHA-derived RvD5, which, in analogy to LX biosynthesis, also requires the dual action of 5-LOX and 12/15-LOX (Gilbert et al., 2021; Kahnt et al., 2023), was not inhibited by the FLAP antagonists in the same cell. This applies to M2-MDMs, as well as to neutrophils and neutrophil–platelet incubations, exposed to bacterial exotoxins. These data are in contrast to previous findings where, in A23187-activated human neutrophils, formation of RvD1 and RvD5, along with RvE1, was inhibited by MK-886 (Mainka et al., 2022). Moreover, conversion of exogenously added 17-HDHA to RvD1 in neutrophils was blocked by MK886 (Lehmann et al., 2015). The discrepancies between the present findings and the previous results might be explained by the involvement of distinct PLA_2_ isoforms at distinct locales that are recruited in response to the different stimuli, resulting in divergent LM profiles: in A23187-activated cells, cPLA_2_ might be the predominant isoform yielding substantial AA-derived 5-LOX products in conjunction with FLAP at the nuclear envelope but less DHA-derived SPM, while exotoxins may recruit a DHA-liberating PLA_2_, leading to substantial DHA-derived 15-LOX products, including RvD5, distant from FLAP and the nucleus. The strikingly different impact of the three tested FLAP antagonists quiflapon, fiboflapon, and BRP-201 on 7-HDHA formation in SACM-stimulated M2-MDMs is in favor of this hypothesis, as we proposed previously for BRP-201 (Kretzer et al., 2022a). Since in M1-MDMs, the FLAP antagonists (except BRP-201) slightly suppressed 7-HDHA formation, we anticipate that the formation of a small portion of 7-HDHA by 5-LOX occurs at the nuclear envelope, especially in M1-MDMs supported by FLAP, while the majority of 7-HDHA is formed by cytosolic 5-LOX without FLAP, especially in M2-MDMs. Nevertheless, the degree of inhibition of 7-HDHA production is minor compared to the AA-derived 5-LOX products, which strongly depends on FLAP at the nuclear envelope. Notably, whether the low levels of RvD5 in neutrophil–platelet incubations are formed by 12/15-LOX activity is still unclear; further investigations are needed to reveal SPM formation in such coincubations by also considering other cellular effects, such as membrane disruption with a subsequent release of 7- or 17-HDHA from phospholipids by phospholipases.

Previous findings with BRP-201 that induced 15-LOX product formation in resting M2-MDMs raised the question of whether FLAP antagonism is related to 15-LOX activation and induction of SPM biosynthesis (Kretzer et al., 2022a). Although the pattern of the LM-modulatory effect of all compounds was comparable in activated cells, only quiflapon, BRP-7, BRP201, and MK886, which are somehow structurally related benzimidazole- or indole-based compounds, caused the induction of 15-LOX product formation in M2-MDMs and, thus, increased RvD5 levels. Therefore, we suggest that FLAP antagonists with indole or benzimidazole moiety can have an inducing effect on RvD5 biosynthesis while other FLAP antagonists do not. Notably, structurally diverse compounds devoid of FLAP antagonistic features, such as 3-*O*-acetyl-11-keto boswellic acid, celastrol, or chalcones, can induce 15-LOX activity and SPM production in MDMs, monocytes, or neutrophils (Pace et al., 2021; Kretzer et al., 2022b; Borner et al., 2023). Possibly, BRP-201, as well as quiflapon, BRP-7, and MK886, also acts on 15-LOX, potentially at an allosteric site between the catalytic and the C2-like domain, where AKBA also binds and mediates its stimulatory effect (Borner et al., 2023), although chemical structural similarities are not readily apparent between AKBA and the FLAP antagonists.

In conclusion, several FLAP antagonists were developed in the last three decades and have been intensively evaluated for the therapy of asthma, COPD, arthritis, and cardiovascular disease in clinics, with partially promising results (Gur et al., 2018a; Prescott et al., 2022). However, no FLAP antagonist has yet reached the market for multiple reasons, in particular, due to their low bioavailability related to strong plasma protein binding (Gur et al., 2018a). Our data from the comparative analyses of the impact of the different FLAP antagonists on LM profiles in biologically relevant test systems provide important implications for the pharmacology and application of a FLAP-directed strategy in pharmacotherapy. All compounds combine potent suppression of pro-inflammatory LT and anti-inflammatory LX formation, with not only beneficial (LT suppression) but also detrimental functions (LX suppression). Obviously, the requirement of FLAP for 5-LOX-mediated SPM formation depends on the type of PUFA substrate, where FLAP provides AA for LX generation, while DHA conversion by 5-LOX to RvD5 proceeds without FLAP. The failure of FLAP antagonists to block RvD5 formation is encouraging and might be advantageous compared to 5-LOX inhibitors that block the production of D-series RVs (Werner et al., 2019).

## Data Availability

The original contributions presented in the study are included in the article/Supplementary Material, further inquiries can be directed to the corresponding authors.
