# Supplementary material for: Differential impact of 5-lipoxygenase-activating protein antagonists on the biosynthesis of leukotrienes and of specialized pro-resolving mediators
Source: Front Pharmacol. 2023 Aug 23;14:1219160. doi: 10.3389/fphar.2023.1219160 (PMC10481534; doi:10.3389/fphar.2023.1219160)
Supplement: Supplementary file 1 [file Table1.DOCX]

Supplementary Material

Differential impact of 5-lipoxygenase-activating protein (FLAP) antagonists on the biosynthesis of leukotrienes and of specialized pro-resolving mediators (SPM)

Philipp Dahlke^1^, Lukas K. Peltner^1^, Paul M. Jordan^1,2,*^, Oliver Werz^1,2,*^

*** Correspondence:**Paul M. Jordan and Oliver Werz
[paul.jordan@uni-jena.de](mailto:paul.jordan@uni-jena.de) and [oliver.werz@uni-jena.de](mailto:oliver.werz@uni-jena.de)

**Supplemental Table 1**

**Supplemental Table 1. Lipid mediators produced by neutrophils and neutrophil-platelet coincubations after stimulation with Ca^2+^-ionophore A23187 or with SACM.** Isolated neutrophils (1 × 10^7^ cells/mL) or coincubations of neutrophils and platelets (1 × 10^7^ and 25 × 10^7^ cells/mL) were incubated in PBS pH 7.4 containing 1 mM CaCl_2_ with SACM (1%) or Ca^2+^-ionophore A23187 (2.5 µM) for 90 min or 15 min, respectively. Results are given as mean ± SEM, n = 7; n.d. = not detectable.

**Supplemental Figure 1**


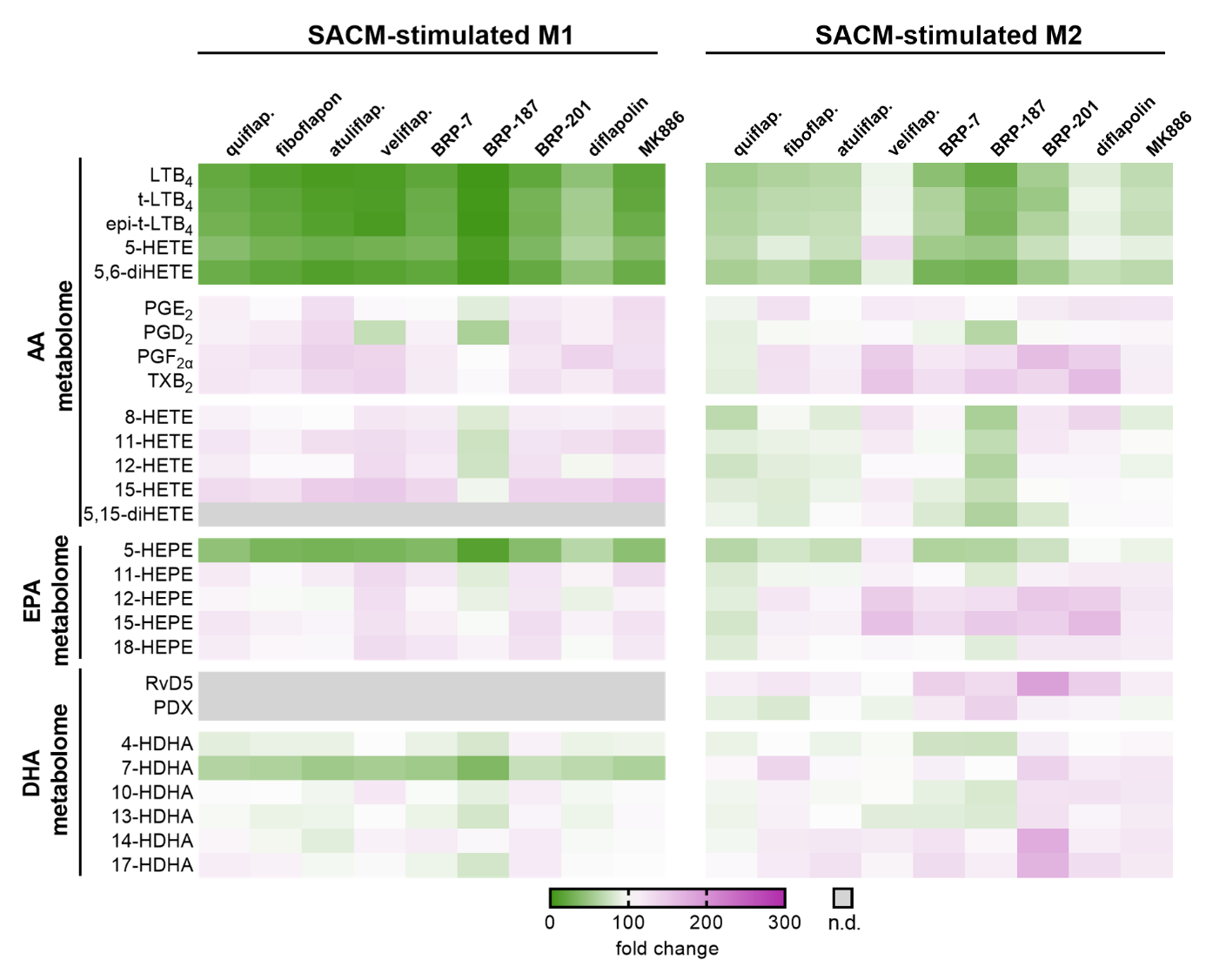


**Supplemental Figure 1. Modulation of agonist-induced lipid mediator formation by FLAP antagonists in monocyte-derived macrophages.** Human M1- or M2-MDM (0.5 × 10^6^ cells) were preincubated with the indicated FLAP antagonists quiflapon, fiboflapon, atuliflapon, and MK886 at 0.03 µM, veliflapon, BRP-187, BRP-201 and diflapolin at 0.3 µM, and BRP-7 at 1 µM, or with vehicle (DMSO, 0.1%) for 15 min before challenge with SACM (1%) for 90 min at 37 °C. Formed LM were quantified in the supernatants using UPLC-MS-MS. Data (n = 3) are given as heatmaps presenting the -fold change to SACM-stimulated vehicle control.

**Supplemental Figure 2**


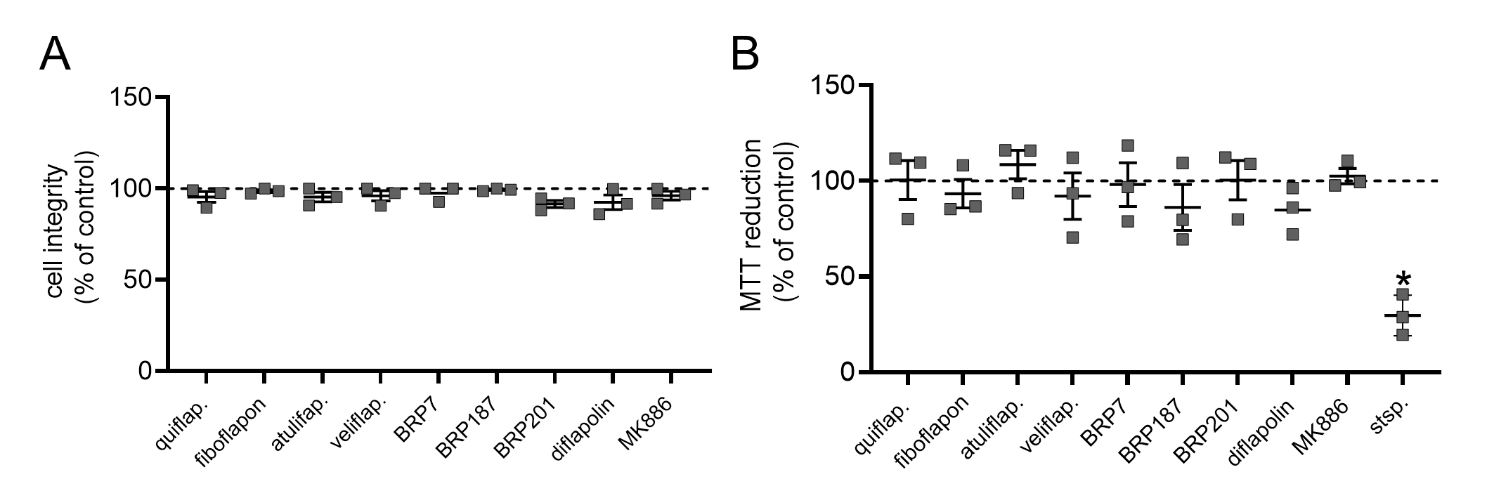


**Supplemental Figure 2. FLAP antagonists do not induce cytotoxicity.** Human unpolarized MDM were kept in RPMI 1640 medium and incubated with the indicated FLAP antagonists quiflapon, fiboflapon, atuliflapon, BRP-7 and MK886 at 1 µM, each, and veliflapon, BRP-187, BRP-201 and diflapolin at 3 µM, or with vehicle (0.1% DMSO) for 3 h for LDH assay (**A**) and 48 h for MTT assay (**B**). For MTT assay, staurosporine (stsp., 1 µM) as positive control was used. Shown are means ± SEM with single values, given as percentage of control (DMSO = 0.1%). *n* = 3. For statistical analysis data were log-transformed and analyzed via one-way ANOVA and Dunnett’s multiple comparison test. * p < 0.05.

**Supplemental Figure 3**


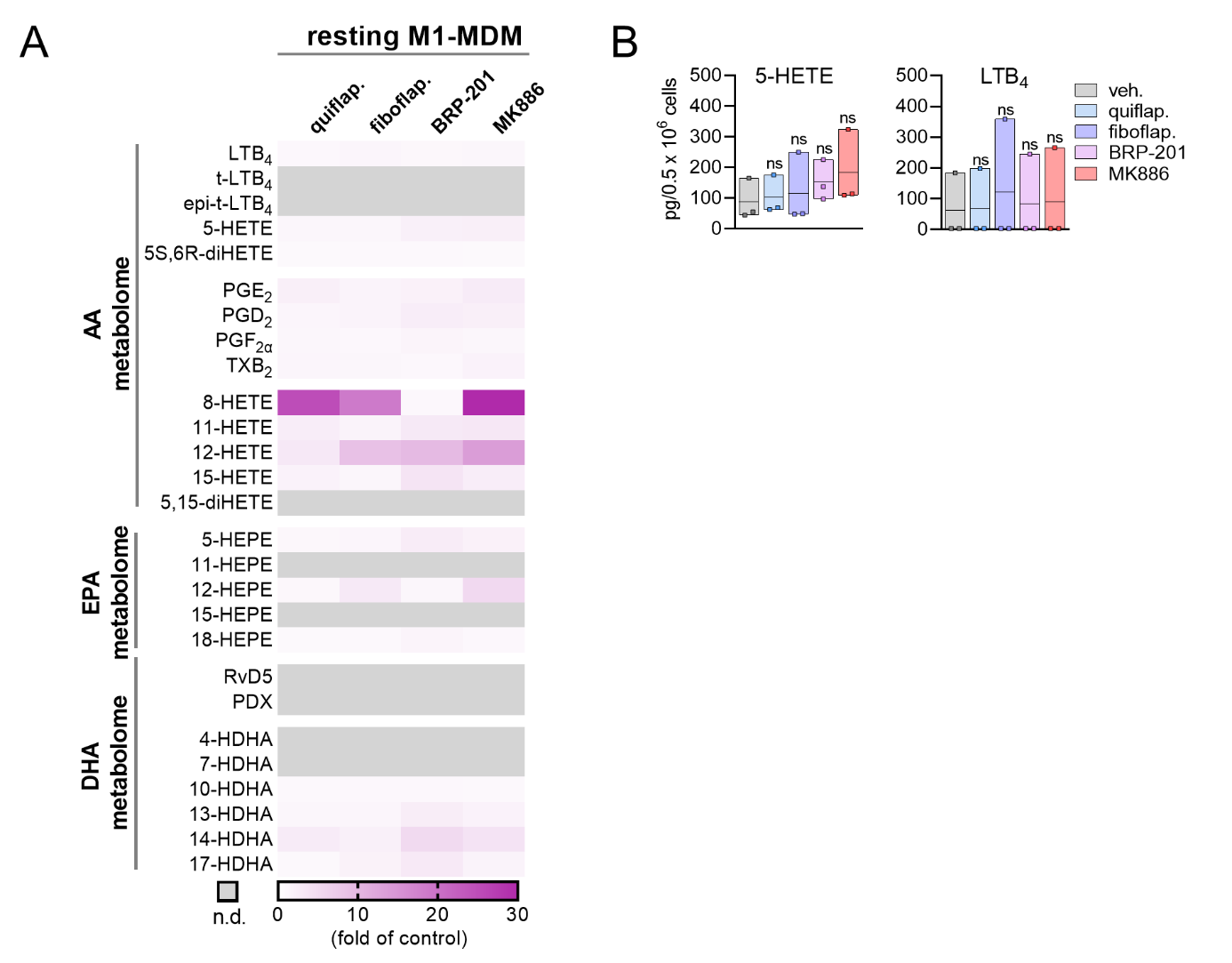


**Supplemental Figure 3. Induction of LM formation by FLAP antagonists in M1-MDM.** (**A,B**) Human M1-MDM (0.5 × 10^6^ cells) were incubated with FLAP antagonists BRP-7 at 10 µM, quiflapon, fiboflapon, atuliflapon, and MK886 at 1 µM and veliflapon, BRP-187, BRP-201 and diflapolin at 3 µM or vehicle (DMSO, 0.1%) for 180 min and formed LM were quantified in the supernatants using UPLC-MS-MS. Results are given as a heatmap presenting the fold change to vehicle control in (**A**) and in (**B**) data for 5-HETE and LTB_4_ are given as single values and means in floating bar charts; n = 3. For statistical analysis data were log-transformed and analyzed via matched one-way ANOVA with Dunnett’s multiple comparison test. * p < 0.05, ** p <0.01.

**Supplemental Figure 4**
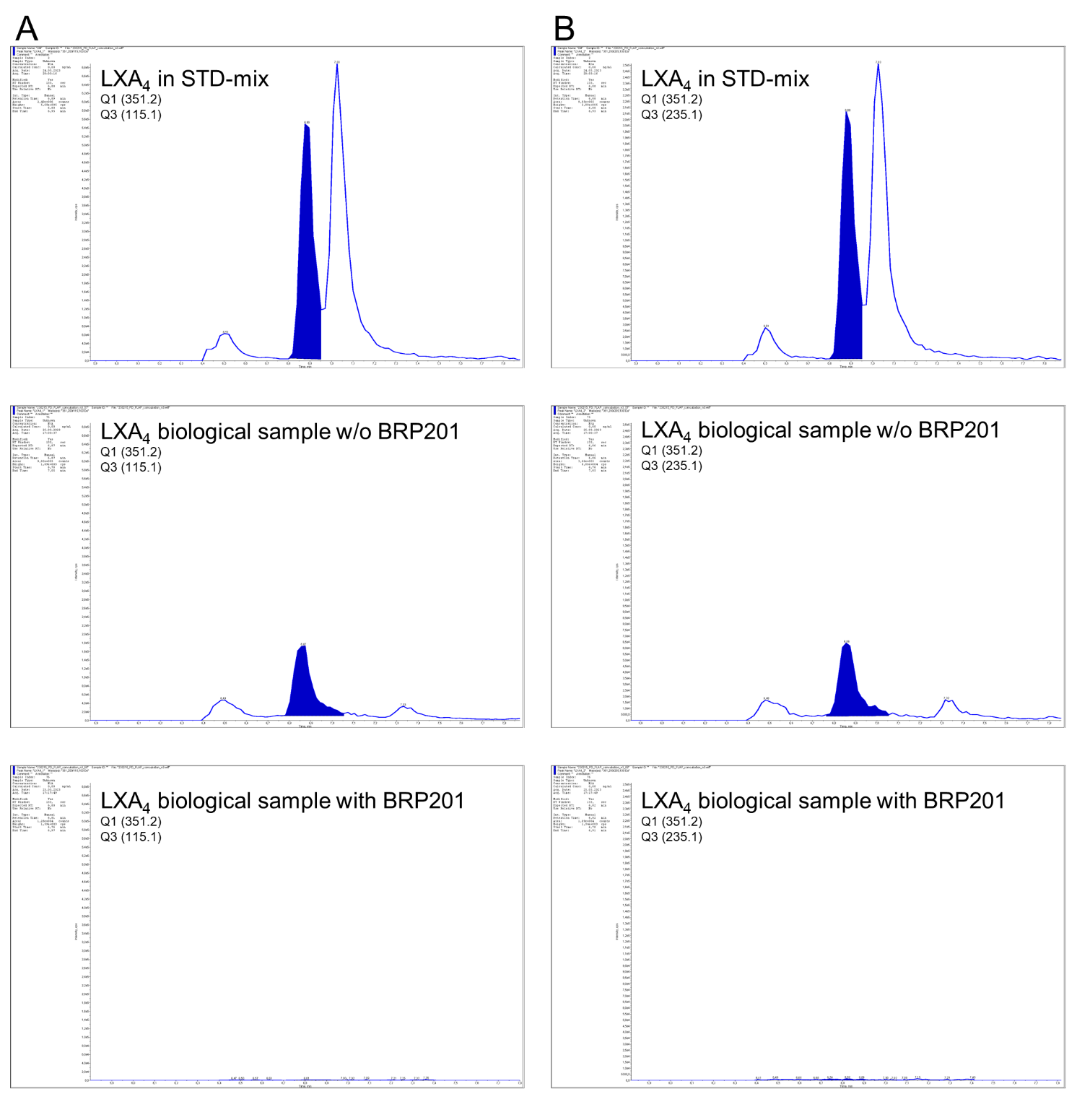


**Supplemental Figure 4. Verification of LXA_4_ in biological samples.** Selective ion monitoring (SIM) of LXA_4_ in (**A**) for Q1:351.2 and Q3:115.1 and in (**B**) for Q1:351.2 and Q3:235.1 in the standard mix (STD-mix), as well as in a representatives samples of A23187-stimulated neutrophil and platelets coincubation with or without preincubation with BRP-201 (data shown in **Figure 3B,C**).
