# Supplementary figures and images for: Differential impact of 5-lipoxygenase-activating protein antagonists on the biosynthesis of leukotrienes and of specialized pro-resolving mediators
Source: Front Pharmacol. 2023 Aug 23;14:1219160. doi: 10.3389/fphar.2023.1219160 (PMC10481534; doi:10.3389/fphar.2023.1219160)

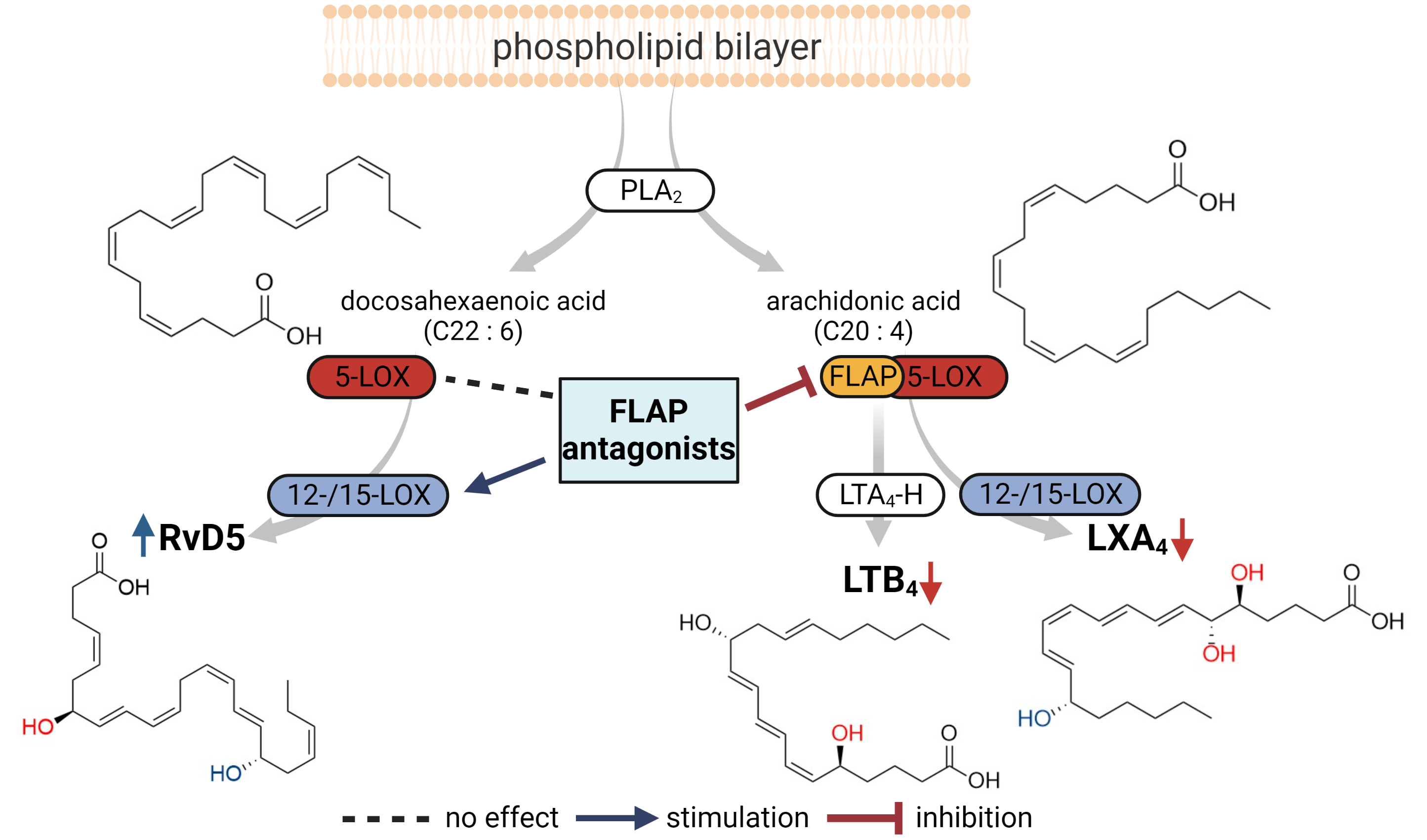

Supplement: Supplementary file 2 [file Image1.PNG]
